# Alcohol and dementia: is hepatic steatosis an innocent bystander or an active contributor to cognitive decline?

**DOI:** 10.1007/s12072-026-11149-6

**Published:** 2026-09-09

**Authors:** Cameron Gofton, Elie Matar, Jacob George

**Affiliations:** 1 Northern Clinical School, Faculty of Medicine and Health, University of Sydney, Sydney, Australia; 2 Department of Gastroenterology and Hepatology, Royal North Shore Hospital, Sydney, NSW Australia; 3 Storr Liver Centre, The Westmead Institute for Medical Research and Westmead Hospital, University of Sydney, Sydney, NSW 2145 Australia; 4 Central Clinical School, Faculty of Medicine and Health, University of Sydney, Sydney, Australia; 5 Centre for Integrated Research and Understanding of Sleep (CIRUS), Woolcock Institute of Medical Research, Sydney, NSW Australia; 6 Department of Neurology, Royal Prince Alfred Hospital, Sydney, Australia; 7 Department of Gastroenterology and Hepatology, Westmead Hospital, Sydney, NSW Australia

Recent studies suggest an association between cognitive impairment and the early stages of liver disease that is distinct from the known neurocognitive impacts of cirrhosis or basal ganglia copper accumulation in Wilson’s disease [1, 2]. However, the association between an increased risk of cognitive impairment following chronic alcohol excess even after significant abstinence, and the associations between steatotic liver disease (SLD) and the development of various types of dementias including Alzheimer’s disease (AD) and vascular dementia (VD) have been less characterised [1, 2]. A number of plausible mechanisms that could link SLD with cognitive disease have been postulated (Fig. 1). Increasing insulin resistance in SLD has been associated with accumulation of advanced glycated end products, inhibition of intracellular degradation of amyloid-beta, and glucose neurotoxicity, all of which can lead to cognitive impairment. The development of lipid abnormalities associated with SLD can also lead to atherosclerotic vascular injury and chronic low grade systemic inflammation leading to oxidative stress and neuroinflammation which impacts cognitive resilience leading to impairment. Whilst these mechanisms could link SLD with cognitive decline, a clear causal relationship has not been demonstrated, particularly in the context of alcohol use [3].

**Fig. 1:**
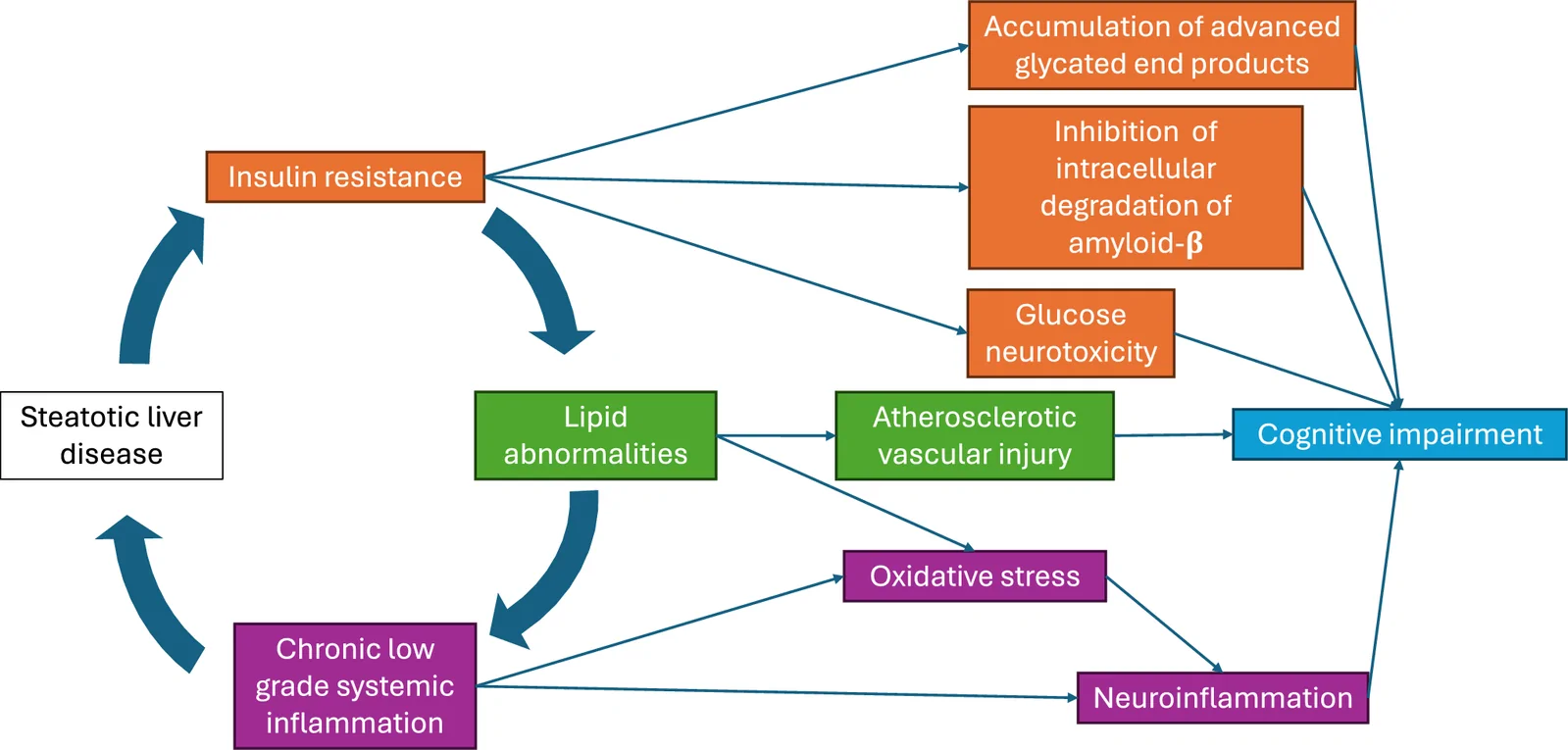
Proposed mechanisms of cognitive impairment in steatotic liver disease

In this issue of the Journal, Han and colleagues sought to understand this relationship utilising the Korean National Health Insurance Service (NHIS) data of individuals who underwent health examinations in 2012. Participants were classified into those with and those without metabolic dysfunction-associated SLD, metabolic dysfunction-associated alcohol-related liver disease (MetALD), and alcohol-related liver disease (ALD). The metabolic liver disease group underwent subgroup analysis with individuals classified as non-drinkers (no alcohol) and individuals with within-threshold alcohol intake of ≤20 g/day for Female and ≤30 g/day for male (MASLD-WTAI). Of 3,071,829 adults surveyed in 2012 without a pre-existing diagnosis of dementia, 72,425 developed dementia over a median follow up of 5.4 years. The risk of dementia increased across all dementia outcomes in SLD, when compared to those individuals without SLD (aHR, 1.21, 95% CI 1.18–1.25 for all-cause dementia; aHR,1.19; 95% CI 1.15–1.23 for AD; and aHR, 1.25; 95% CI 1.17–1.34 for VD).

Across SLD subtypes, as one would expect, ALD showed the strongest positive association with dementia (aHR 1.67; 95% CI 1.54–1.81 for all-cause dementia; aHR 1.65; 95% CI 1.50–1.82 for AD; and aHR 1.52; 95% CI 1.25–1.85 for VD). SLD also demonstrated positive associations with all types of dementia (aHR 1.19; 95% CI 1.16–1.23 for all-cause dementia, aHR 1.18; 95% CI 1.14–2.33 for AD, and aHR 1.23; 95% CI 1.14–1.33 for VD). Of interest, MetALD demonstrated largely neutral associations but with a modest association with VD (aHR 1.23; 95% CI 1.02–1.48 for VD). In a subgroup analysis of MASLD by alcohol consumption, the MASLD-no alcohol group showed an increased association with all dementia outcomes (aHR 1.29; 95% CI 1.25–1.34 for all-cause dementia, aHR 1.28; 95% CI 1.24–1.33 for AD, aHR 1.30; 95% CI 1.19–1.43 for VD). Compared to this reference, MASLD-WTAI demonstrated no association with all-cause dementia but a modestly increased risk of VD (aHR, 1.12; 95% CI 1.01–1.26).

This analysis used a very large database comprising ~ 10% of the Korean adult population. The strongest signal of an increased dementia risk in ALD is biologically coherent with known literature on the effects of alcohol on cognitive function with contributing physiological effects from alcohol-related neurotoxicity, vascular injury, nutritional deficiency, neuroinflammation, sleep disruption, and probably, reduced brain resilience in the setting of systemic/liver disease [4]. This study further hints that SLD has a causal association with the development of cognitive impairment.

However, there are several caveats to this interpretation. One issue with all types of big data analyses is the input data variables. For this study, fatty liver was diagnosed using the fatty Liver Index, which is not as robust as imaging-based diagnosis, with MRI-PDFF being the most robust. Similarly, the use of coding data to exclude other liver diseases and to diagnose dementia introduces potential misclassification bias.

The present study also had a short median follow-up time of 5.4 years. Neurocognitive studies establishing associations to dementia often have long latency, with research suggesting that the time for change in cognitive abilities to diagnosis of mild cognitive impairment is between 3 and 7 years, whilst from mild cognitive impairment to dementia it is another 1–11 years [5]. Whilst the authors attempted to mitigate this by utilising time-lag analysis of 2 and 3 year periods to exclude those individuals with early diagnosis of dementia, it cannot fully adjust from the known natural history and prolonged prodromal trajectory of dementia.

As with every retrospective analysis, when it comes to alcohol intake, there are concerns regarding the veracity of the data collected and the derived outcomes from the analysis. Alcohol history is notoriously prone to under-reporting. The Korean NHIS questions to determine alcohol consumption patterns were “On an average, how many days per week do you drink?”, and “On days when you drink, how much do you typically consume in a day?”. Based on these questions, there is a potential classification bias in terms of current drinkers, former drinkers, and non-drinkers. This is highlighted by the fact that 9.1% of those diagnosed ALD were classified as non-drinkers. Whilst these individuals had previous significant alcohol intake in their prior history, this was not captured in the data that was collected. The assumption that alcohol intake from the initial assessment in 2012 did not change until study analysis is also flawed as alcohol use is known to be highly variable across the life course.

Of interest, the association with MASLD-WTAI and dementia decreased with longer lag periods, suggesting that this effect is modest at best. The signal for the association weakened after excluding early dementia cases and therefore argues against a durable protective effect. Rather, this finding raises the possibility of reverse causation, whereby individuals with MASLD-WTAI who developed dementia earlier may already have been in the prodromal dementia phase, or in poorer health at baseline, leading to reduced alcohol consumption prior to study enrolment. Coupled with the E-value of 1.07 presented in this study which quantifies robustness of relationship against unmeasured confounding, it suggests that the MASLD-WTAI association is the weakest relationship and the one most susceptible to residual confounding.

The utilisation of a MASLD-no alcohol group as a clean healthy reference group needs to be interpreted with caution. There is a known bias in both alcohol and dementia epidemiology of reverse causation, sick quitter/abstainer bias, and healthy user effects that can skew study results, particularly in large datasets lacking sufficient granularity [6]. Individuals with poorer health may be more likely to abstain from alcohol consumption and thus be classified as non-drinkers, as highlighted by the non-drinker group in the ALD group mentioned above. In contrast, individuals who continue to drink modest amounts of alcohol may represent a healthier or socially more advantaged group, associated with other neuro-protective behaviours and factors such as higher education or socioeconomic status (e.g. so-called “fancy wine drinkers”). This could also explain the mostly neutral effects of MetALD on dementia prevalence demonstrated in this study. These biases have been more recently brought to light in relation to studies examining alcohol consumption and dementia, including in large cohort, case–control, and Mendelian randomisation analyses [6]. Whilst Han and colleagues tried to address this within the constraints of the data collected, any inference regarding alcohol consumption and its protective effects should be interpreted with restraint.

From a neurological perspective, historical dementia subtypes have become more nuanced over the years. Administrative AD and vascular dementia diagnoses by ICD-10 codes are useful in large scale epidemiological studies but are not biological diagnoses. The presence of alternative or co-pathology dementias including vascular brain injury, cerebral amyloid angiopathy, Lewy body disease, mixed pathology or other contributors undoubtedly clouds the assessment of the associations in this study. In the dementia space there has also been a significant shift towards biomarker driven diagnoses, particularly for AD, which would assist in untangling these dementia subtypes, although such data was not available in this study [7]. It is also important to recognise that vascular dementia diagnosis is often applied inconsistently. Epidemiological estimates vary considerably depending on whether vascular dementia is defined narrowly as cognitive decline temporally related to a cerebrovascular event (estimated as 2% in recent studies [8]), or more broadly as cognitive impairment in the context of vascular brain injury and associated radiological changes. In this light, the relatively high proportion of vascular dementia diagnoses amongst individuals with SLD in this study of ~16% (Table 4 of Han et al.), may partly reflect the enrichment of this cohort for vascular and metabolic risk factors, making vascular cognitive impairment biologically plausible. This should still be interpreted carefully as administrative coding may also capture broader vascular contributions to cognitive impairment rather than clinically adjudicated pure vascular dementia.

An area that may have improved the delineation between any association of SLD and the development of dementia would have been classification of the severity of liver disease. Whilst more robust measures of assessing steatosis and fibrosis were not available to test associations between these and vascular and Alzheimer’s dementia, blood biomarkers such as FIB-4 or the NAFLD Fibrosis Score could have been utilised. Demonstrating a gradient in dementia risk with fibrosis stage (a useful surrogate of metabolic multi-morbidity) would suggest a more plausible causative link, since there are many confounders for any association between steatosis and dementia risk.

This study highlights an important and exciting area of research, with several plausible biological mechanisms linking SLD, alcohol exposure, metabolic dysfunction, and cognitive impairment, as summarised in the Fig. 1. Whilst this research is premature to speculate on potential approaches to cognitive screening in patients with SLD and alcohol-related liver disease, it does suggest that this should be considered for earlier implementation in clinical practice. To effectively establish these relationships, prospectively designed studies with detailed longitudinal biomarker assessments such as MRI-PDFF with fibrosis assessments are needed. Due to the long follow-up required and the lack of liver-specific biomarker assessment, this study adds to the literature but highlights that more work is needed to definitively answer the question of whether SLD is an innocent bystander or is causally involved in cognitive impairment.

## Data Availability

Not applicable.
